# Supplementary material for: OmniSegger: A time-lapse image analysis pipeline for bacterial cells
Source: PLoS Comput Biol. 2025 May 28;21(5):e1013088. doi: 10.1371/journal.pcbi.1013088 (PMC12140430; doi:10.1371/journal.pcbi.1013088)
Supplement: S1 Text — (PDF) [file pcbi.1013088.s001.pdf]

Supplementary Material: OmniSegger

CONTENTS

A. Feature updates: From SuperSegger to OmniSegger 1
1. Omnipose segmentation 1
2. OmniSegger's automatic error correction reduces fatal errors 1
3. Improvements in data visualization 2
4. Modified cell length measurement 2
5. Improvements in accessibility 3
a. The clist as an Excel spreadsheet 3
b. ND2 to TIFF conversion 3

B. Limitations of OmniSegger 3
1. Discussion: Challenges for linking algorithms 3
2. Results: Bactrack–An alternative linking algorithm 4
3. Results: Modular segmentation 4
4. Discussion: Generalization of cell cytometry software 5

C. Proliferation challenge: Cell width measurement 5

D. Training the brightfield Omnipose model 5

E. Materials and Methods 6
1. Bacterial sample preparation 6
2. Microscopy methods 6
3. Package versions 6
4. Optimal frame rate for imaging 7
5. Reducing segmentation errors using frame skip. 7
6. Reducing segmentation errors using mask registration. 7
7. Computational resources 7
8. Computational runtime 7
9. Image processing 8

Appendix A: Feature updates: From SuperSegger to OmniSegger

OmniSegger skips the original SuperSegger cell segmentation algorithm, and runs the Omnipose package. The SuperSegger mask variables are replaced by the masks output by Omnipose. Omnipose segmentation is far more robust to diverse cell morphologies in addition to improving performance on a subcellular scale. As a result, Omnipose provides the foundation for time-lapse analysis by OmniSegger. In addition, the data visualization features have also been updated to accommodate improved segmentation results.

1. Omnipose segmentation

Omnipose is a deep neural network (DNN)-based algorithm for cell segmentation [3]. It is originally based off of the Cellpose algorithm [24]; however, it makes several changes which significantly improve performance.

The use of the distance field, suppressed Euler integration, and training a phase-contrast model on a diverse dataset allows Omnipose to be a leading segmentation tool. Omnipose is much more robust to various imaging conditions and cell morphologies than other cell segmentation algorithms [3].

2. OmniSegger's automatic error correction reduces fatal errors

OmniSegger performs automatic error correction during the linking step. (Although this error correction can be turned off, as described in the documentation. We do not recommend this.) It can correct some 1-to-2-to-1 (and 2-to-1-to-2) tracking errors by fusing (fissioning)

the errant intermediate-frame mask. If the error is automatically corrected and leads to a successful linking (after the correction), the error is not a "fatal error" and is therefore not counted in Fig. 2C. For the highest frame rates, we recommend turning on the "frame skip", which segments "key frames" (by skipping a user-specified number of frames) and then copies these key-frame masks to the unsegmented frames. See Appendix E 5.

3. Improvements in data visualization

We introduce new data visualization ideas and improvements to generalize for diverse cell morphologies: 1) cell outlines, 2) medoid for cell ID display, and 3) figure making tools.

1. **Cell outlines.** The displayed cell outlines were previously calculated by dilating the masks. The outlines are now calculated using a more robust cell perimeter function directly based on the mask (`getperim.m`).

2. **Medoid of cell skeleton for cell ID display.** Many cell analysis software packages, including the original SuperSegger, determine the centroids of cell masks as an approximation for the cell 'center' and use the centroid in order to perform measurements. A centroid is a point calculated from the mean position of all data points, *i.e.*, the mean pixel position of the cell mask. For a given number of N pixels with the coordinate of pixel i given by x_i, y_i, the centroid $(\bar{x}, \bar{y})$ is simply calculated by:

$$\bar{x} = \frac{\sum_i x_i}{N} \tag{A1}$$

and

$$\bar{y} = \frac{\sum_i y_i}{N} \tag{A2}$$

In MATLAB, this is calculated using the `regionprops` function.

The centroid approximation for the cell 'center' fails for more diverse morphologies, for example with filamented cells that contain curvature, where the centroid would fall outside the mask. Rather than calculating the centroid, OmniSegger determines the *medoid*. The medoid is the position of a pixel contained in the cell mask which has the minimum sum of distances from every other pixel in the mask:

$$\min d_i = \min \sum_{j \neq i} \sqrt{(x_j - x_i)^2 + (y_j - y_i)^2} \tag{A3}$$

Furthermore, restricting the medoid to the skeleton results in an even more intuitive result for the cell 'center' (see `find_medoid.m`). Using the medoid of the skeleton as the cell 'center' displays the cell IDs much more intuitively than the centroid, applicable to both rod-shaped and other morphologies.

3. **Figure making tools.** Generating publication-quality figures is important for disseminating results of scientific research. OmniSegger includes new functions for generating figures: `getFamily`, which determines a cell lineage from a progenitor cell, `drawCellSpline`, which draws vectorized outlines of cell boundaries on a cell-by-cell basis, and `makeMosaic`, which generates a mosaic image of selected frames from a time-lapse, with the ability to display fluorescence and cell outline overlays.

4. Modified cell length measurement

In SuperSegger, the cell length is calculated using MATLAB's `regionprops` function. `regionprops` fits a cell as an ellipse, and we expect this approximation to be poor for morphologies with curvature. Therefore, we replaced the cell length measurement with a rod length in `rodGeom`.

The area A of a cross-section of a 3D rod is approximated by a rectangle of length L and width $2R$ with two half-circle end caps of radius R:

$$A = \pi R^2 + 2LR \tag{A4}$$

We can define the length of this rod as the length of the rectangle plus with the two end caps:

$$\ell \equiv L + 2R \tag{A5}$$

We can also find the integral of the distance field, $B = B_{\text{caps}} + B_{\text{rect}}$:

$$B = \int_0^R (R - r) 2\pi r dr + \int_0^R (R - r) 2L dr \tag{A6}$$

and evaluate to find:

$$B = \frac{\pi}{3} R^3 + LR^2 \tag{A7}$$

As we want to solve for the rod radius R and length ℓ, we substitute for L in Eqs. A4 and A7 using Eq. A5 to find:

$$A = (\pi - 4)R^2 + 2\ell R \tag{A8}$$

$$B = \frac{\pi}{3} R^3 + (\ell - 2R)R^2 \tag{A9}$$

We first solve for R. We combine the equations for A and B to cancel out the terms with ℓ resulting in a cubic

equation, which we rearrange to the form of a depressed cubic equation:

$$R^3 - \frac{3A}{\pi}R + \frac{6B}{\pi} = 0 \tag{A10}$$

We define two constants p and q to solve the depressed cubic equation:

$$p \equiv -\frac{3A}{\pi} \tag{A11}$$

$$q \equiv \frac{6B}{\pi} \tag{A12}$$

We then calculate a quantity related to the discriminant:

$$D_1 = \frac{q^2}{4} + \frac{p^3}{27}, \tag{A13}$$

and using Cardano's formula, calculate the three possible roots for R:

$$C_1 = \sqrt[3]{-\frac{q}{2} + \sqrt{D_1}} \tag{A14}$$

$$C_2, C_3 = C_1 * \frac{-1 \pm \sqrt{-3}}{2} \tag{A15}$$

The resulting values are then filtered for only real, positive roots, and the smallest root is chosen as the *rod radius*, r_{rod}. The *rod length*, ℓ_{rod}, is then calculated by plugging in the rod radius into Eq. A4:

$$\ell_{\text{rod}} = \frac{A - \pi r_{\text{rod}}^2}{2r_{\text{rod}}} + 2r_{\text{rod}} \tag{A16}$$

In MATLAB, we calculate B using the discrete sum (rather than the integral) of the distance field of the mask. To account for this discrepancy, we subtract an offset term found from simulating pre-defined masks and calculating their rod radius and length:

$$B' = B - 0.5A_{\text{mask}} \tag{A17}$$

B' is used when calculating q in Eq. A12.

5. Improvements in accessibility

a. *The clist as an Excel spreadsheet*

To improve the accessibility and ease of analysis for researchers, we include a new function `clist2xls.m` which allows users to save the clist as an Excel sheet, in addition to its default `.mat` format.

b. *ND2 to TIFF conversion*

We include a Python script `nd2totiff.py` which utilizes the `aicsimageio` package to save ND2 files as individual TIFF files with the OmniSegger naming convention. The script can accommodate multiple channels, XY positions, time-points, and z-planes; in addition, metadata is saved as a TXT file.

Appendix B: Limitations of OmniSegger

While OmniSegger offers significant improvements to the time-lapse analysis pipeline, in practice, various issues remain. The most pressing issues for OmniSegger at the moment are i) segmentation errors and inconsistent calling of divisions possibly causing cell linking errors, and ii) an open question about the capabilities of cell segmentation-analysis software packages.

1. Discussion: Challenges for linking algorithms

The majority of cell tracking algorithms–including OmniSegger–take masks as inputs, then define frame-to-frame linking costs for each cell mask based on properties such as mask overlap, and minimize the costs to determine links. Determining links and segmentation simultaneously is often computationally intensive, as the cost matrix presents an exponentially growing combinatorics problem; if not determined simultaneously, cell tracking accuracy is totally dependent on cell segmentation results. Thus, if the preceding cell segmentation contains errors, the tracking and lineage determination will be disrupted.

Precisely determining the exact time of division for a cell in an experiment is impossible–time-lapses observe the instantaneous event in discrete time steps. Furthermore, the time of division is often ambiguous when only observed by phase-contrast imaging; this was the primary reason for training the Omnipose bacteria phase-contrast model based on underlying membrane or cytosol fluorescence signal when possible. Due to the ambiguous nature of the phase-contrast image, the algorithm will have a 'flickering' effect; for example, the cell may be determined to be divided into two cells in frame t_0, but one cell in the subsequent frame $t_0 + 1$, and back to two cells in frame $t_0 + 2$. This flickering effect presents an issue for 2D segmentation algorithms, especially when the time-lapses are taken at high frame rate. At the moment, existing 2D segmentation models only perform segmentation by considering individual frames, while the model determination about cell division must persist across multiple frames.

Omnipose introduces the idea of a 'spacetime' model, where the segmentation algorithm takes temporal information into account, effectively becoming a 3D model (2D+T). Let's consider the kymograph for a cell dividing

into two daughter cells: the mother cell grows until the cell wall septates roughly near the center. Conceptually, the kymograph appears like pants; as time increases, the mother is the waist of the pants, which splits into two daughter pants legs. The split is persistent in time. Assuming a frame rate high enough that cells overlap from frame to frame, the 3D spacetime segmentation also presents a solution for cell linking, as each 3D lineage volume contains the time of division and the mother-daughter information. While the 3D spacetime Omnipose model (`bact_phase_spacetime`) is promising, it is lacking the large ground-truth, annotated training dataset as used for the 2D segmentation model and as a result is much less robust.

Though highly robust, Omnipose's `bact_phase_omni` used with OmniSegger can have segmentation errors and in addition, its 2D model does not fix the persistent cell division issue.

A unique feature of SuperSegger/OmniSegger is the inclusion of error resolution functions which can edit the underlying masks to solve segmentation and linking errors, though it is also not fully robust. For further detail on this error correction method, see Appendix E 6 in the Materials and Methods.

2. Results: Bactrack–An alternative linking algorithm

As previously mentioned, there are various limitations to the original tracking algorithm. We have implemented a form of the *Bactrack* package into OmniSegger, which is offered as an alternative option for cell tracking, specializing in linking for diverse morphologies.

Motivated by observations where the original linking algorithm performed poorly for filamentous cells, Sherry Yang developed the Bactrack package [25] in Python, which provided much improved tracking performance. Bactrack is a cell tracking tool which uses hierarchical segmentation and mixed-integer programming (MIP) optimization. The hierarchical segmentation approach of Bactrack was inspired by ultrack [26]; Omnipose flow field inputs are used to create hierarchical segmentations from low to high resolution.

Given a graph of possible segmentations, a matrix of linking costs, and the constraint that cells only divide, Bactrack optimizes the final graph of cell segmentations, effectively solving a MIP problem where it determines the number of cells per frame and therefore also the division times. Bactrack outputs *both* masks in Omnipose labeled-mask format, and linking results as a Pandas dataframe.

To solve the mathematical optimization, Bactrack allows for the option to use one of three different MIP solvers: HiGHS [27], and CBC [28] and Gurobi [29] through Python-MIP [30], though Gurobi and HiGHS are the fastest [31]. OmniSegger only implements HiGHS and Gurobi.

While in theory, the masks that result from the optimized hierarchical segmentation should be more accurate than Omnipose segmentation, in practice, the resulting Bactrack masks were observed to be less biologically accurate; the Bactrack masks often called division too early or too late compared to Omnipose masks when tested on the same dataset. Therefore, the OmniSegger implementation inputs Omnipose masks into Bactrack to generate only linking results. Furthermore, the error resolution code in OmniSegger relies on checking linking results. If the linking results are now determined by Bactrack, error resolution by OmniSegger is not possible and thus the version of OmniSegger with Bactrack implementation does not correct the underlying masks. This can be most dramatically demonstrated by a high time-resolution timelapse, which can suffer from inconsistent determination of division from frame-to-frame.

The linking results contain the mappings between a 'source' (t) frame and a 'target' ($t+1$) frame. The format of results is as follows: the first column lists the source frame starting at frame 0. The second column lists the label IDs in the source frame that have mappings to the target frame. The third column contains the label IDs in the target frame that are mapped from the source frame. In addition, the fourth and fifth columns are source frame cell areas and target frame cell areas, which are used for tracking error calculations. Note that if a cell in a source frame does not have a mapping in the target frame (*i.e.*, a stray cell which appears in the source frame but disappears in the target frame), there will be no entry for that cell ID in the Bactrack linking results; OmniSegger fills these links in `fillBactrackLinks.m`.

The linking results are then converted from Pandas dataframe into comma-separated values (csv). The csv file is read into MATLAB during the linking stage in OmniSegger and then converted into SuperSegger linking format with forward and reverse mappings. In case of errors, the csv can be manually edited before further linking by OmniSegger.

Bactrack is implemented with OmniSegger through the following GitHub branches: Bactrack branch superSeggerDev, OmniSegger branch bactrackdev. This version of OmniSegger is recommended when the main branch of OmniSegger fails to track unusual morphologies for timelapses at medium to low frame rates.

3. Results: Modular segmentation

Omnipose is not fully implemented into OmniSegger. Instead, its output masks is the input to the segmentation step of OmniSegger. In fact, any other segmentation algorithm can be used, as long as the input masks are in `png` format. The modularity of OmniSegger allows it to be compatible with future advances in cell segmentation. Similarly, the *bactrackdev* branch of OmniSegger introduces modularity in linking, as long as the input `csv` is in Bactrack format.

Software	No User-defined Training/Inputs	Segmentation Method	Tracking Method	Visualization GUI	Output Files	Language	OS Support	Quantitation of Non-diffuse Foci	Modality	Automated Plot Generation	Year Updated
OmniSegger	✓	Deep learning (Omnipose)	Traditional	✓	mat, xls	MATLAB & Python	Linux, Windows, MacOS	✓	Phase Pad, Brightfield, Cytoplasmic, Membrane	✓	2024
SuperSegger [4]	✓	ML-informed Threshold	Traditional	✓	mat	MATLAB	Linux, Windows, MacOS	✓	Phase Pad	✓	2018
DeLTA [9]	✓	Deep learning	Deep learning	X	nc	Python	Linux, Windows	X	Phase Pad, Phase MM	X	2024
Ilastik-CellProfiler [10, 11]	X	ML-informed Threshold or Watershed	Traditional	X	xls	Standalone	Linux, Windows, MacOS	X	All: with training	✓	2024
FAST [12]	X	Threshold	Unsupervised learning	✓	mat	MATLAB, Standalone	Linux, Windows, MacOS	X	Phase Pad, Brightfield	✓	2023
CellShape [13]	X	Threshold	N/A	✓	N/A	Python	Linux, Windows, MacOS	✓	Phase Pad	✓	2017
Oufti [14]	X	Threshold	Traditional	✓	mat, out, csv	MATLAB	Linux, Windows	✓	Phase Pad	✓	2016
MicrobeJ [15]	✓	Threshold	Traditional	✓	res, csv	Java (ImageJ)	Linux, Windows, MacOS	✓	Phase Pad	✓	2024

TABLE A. An extended comparison of features and functions for cellular imaging analysis software packages.

4. Discussion: Generalization of cell cytometry software

While the introduction of modular segmentation and linking steps allows OmniSegger to be much more robust to analyzing time-lapses of diverse cell morphologies, the analysis software is still biased towards rod-shaped cells. For example, quantities such as long axis length or cell pole age are calculated. However, consider cocci, which are spherically shaped bacteria. The significance of a long axis or a cell pole measurement becomes unclear for such a morphology. Cytometry calculations must therefore be more generalized, or carefully checked, if analyzing morphologies which are not rod-shaped.

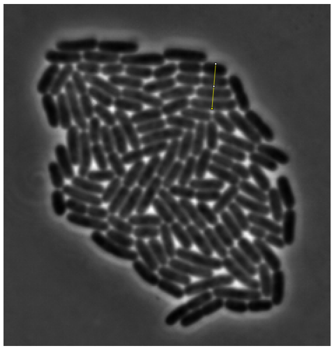

FIG. A. Cell length measurement across four cells from frame 138 of the time-lapse.

Appendix C: Proliferation challenge: Cell width measurement

The cell width used as a proxy for sub-cellular accuracy was estimated in ImageJ by measuring the length of four parallel *E. coli* cells in contact (see Fig. A). The length was then divided by four as an averaging method. We performed this measurement 4 times from frames 121, 133, 138, and 141 of the time-lapse. The average width is estimated to be 0.86 µm.

Appendix D: Training the brightfield Omnipose model

We attempted to train a model to segment brightfield images taken in the focal plane of the bacterial samples. However, we noticed very poor performance of the model upon evaluation of in-focus test data (see Fig. B).

After failing to train a model for images taken in the focal plane, we next attempted to train a model for segmentation of under- and over-focused images. The ground-truth dataset used to train the model does not include in-focus planes, only under- and over-focused planes in increments of 0.1 µm.

Because over-focused brightfield appears similar to phase-contrast, we generated approximate masks using the Omnipose phase-contrast model. Next, the masks were hand-annotated in Napari, informed by membrane-labeled fluorescence signal as validation when the fluorescence images were available, to refine and correct errors. Four to six colors were used to annotate the masks [3]. The masks were then converted to a standard 16-bit integer label mask PNG. The under- and over-focused images had a slight offset which were accounted for by performing a non-rigid image registration on the images with reference to the ground-truth masks.

In addition to our own images, the published brightfield images from DeepBacs [32] were included in the ground-truth dataset.

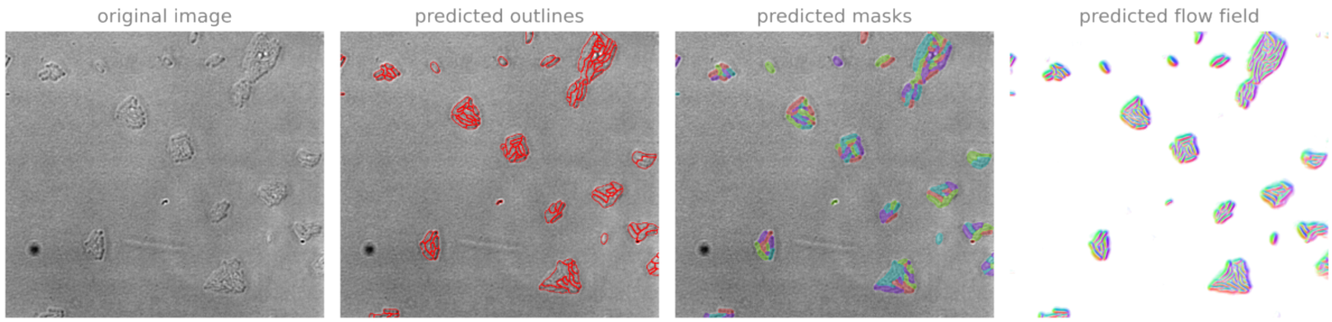

FIG. B. **Brightfield model performance on in-focus image.** Brightfield image of *E. coli* in the focal plane. The low contrast of in-focus brightfield makes the cells difficult to distinguish, both for the human eye and for segmentation algorithms.

Number of images: 471.

Total cell count: 6623.

Further details about the datasets used for ground-truth annotation are included in Data Table S1.

Appendix E: Materials and Methods

1. Bacterial sample preparation

Proliferation dataset: MG1655 *E. coli* was grown in Luria-Bertani media (LB) overnight, then reinoculated into fresh LB medium at a dilution of 1:1000, and grown to OD_{600} 0.1 before imaging. Cells were spotted onto a 4% agarose pad was prepared with M9 minimal media (1X M9 salts, 2 mM MgSO4, 0.1 mM CaCl2, 0.4% glycerol, and 0.2% casamino acids).

Morphology dataset: MG1655 *E. coli* was grown in Luria-Bertani media (LB) overnight at 30°C, then reinoculated into fresh LB medium at a dilution of 1:1000 and grown for 1.5h at 30°C, then pelleted and resuspended in LB with 10μm hydroxyurea and grown for 1.5hr at 30°C. A 2% agarose pad was prepared using the same LB with 10μm hydroxyurea. Cells were spotted onto the pad and left in the microscope chamber at 37°C for 1hr before imaging.

Modality dataset-brightfield: TB28 attHK022 Plac::zipA-sfGFP bla pal-mCherry cat [33] was grown in Luria-Bertani media (LB) supplemented with 100μg/ml ampicillin overnight, then reinoculated into fresh M9 medium at a dilution of 1:100, and grown for 1h at 30°C before imaging. Cells were spotted onto a 2% agarose pad prepared with minimal M9 media (1X M9 salts, 2 mM MgSO4, 0.1 mM CaCl2, 0.2% glycerol, 10 μg/ml thiamine HCl) and left in the microscope chamber at 30°C for 1hr before imaging.

Modality dataset-cytoplasmic fluorescence: ASKA *lysC-GFP* (JW3984) was grown in a 96-deep well plate overnight in Luria-Bertani media (LB) supplemented with 34 μg/mL chloramphenicol (Cm34) at 30°C. The strain was then diluted 1:25 into M9 minimal media (1X M9 salts, 2 mM MgSO4, 0.1 mM CaCl2, 0.2% glycerol, 10 μg/ml thyamine HCl, and 0.2% casamino acids) with Cm34 and allowed to grow to mid-log. Prior to imaging, the fusion expression was induced with 500μM of Isopropyl β-d-1-thiogalactopyranoside (IPTG) for 40min. Cells were spotted onto a 2% agarose pad prepared with M9 media without IPTG [5].

Modality dataset-cytoplasmic fluorescence: ASKA *ygaW-GFP* (JW2645) was grown in a 96-deep well plate overnight in Luria-Bertani media (LB) supplemented with 34 μg/mL chloramphenicol (Cm34) at 30°C. The strain was then diluted 1:25 into M9 minimal media (1X M9 salts, 2 mM MgSO4, 0.1 mM CaCl2, 0.2% glycerol, 10 μg/ml thiamine HCl, and 0.2% casamino acids) with Cm34 and allowed to grow to mid-log. Prior to imaging, the fusion expression was induced with 50μM of Isopropyl β-d-1-thiogalactopyranoside (IPTG) for 40min. Cells were spotted onto a 2% agarose pad prepared with M9 media without IPTG [5].

2. Microscopy methods

Imaging was performed using a Nikon Eclipse Ti-E microscope, through a 60X 1.4 NA CFI oil-immersion Phase objective onto an Andor Neo sCMOS camera. The microscope chamber was heated to 30°C or 37°C.

3. Package versions

The following package versions were used to generate the data used in the figures:

- DeLTA: 2.0.5, main branch, commit 32e75d60; Python 3.11.10
- Ilastik 1.4.0, GPU-enabled
- CellProfiler 4
- SuperSegger: main branch, commit 6d58c6e; MATLAB R2024a
- OmniSegger: main branch, commit 22d9b69; MATLAB R2024a

– Omnipose: main branch, commit a585929; Python 3.10.12

4. Optimal frame rate for imaging

In most contexts, other experimental considerations will set the optimal frame rate; however, we can ask the question what frame rate is optimal for pipeline analysis only. Although this initially seems like a well-posed question, its answer is also experimental-design dependent. To understand this context dependence, it is helpful to explore two mechanisms for pipeline error: segmentation and tracking errors.

Segmentation errors. Let us posit that there is an error probability of e per cell per image of generating a fatal segmentation error. Given a short time interval, the number of frames captured is proportional to the rate and therefore the number of errors is also proportional to the rate. Reducing frame rate therefore reduces segmentation errors.

Tracking errors. Error-free analysis of time lapse data also depends on successful linking of the cells between frames. Our algorithm depends on the overlap between cells in successive frames. The tracking errors become highly frame-rate dependent when analyzing the growth of microcolonies. In log-phase growth, assuming a single layer of cells growing on an agarose pad, the area of the microcolony grows exponentially with the growth rate. This implies that the edges of the microcolony must move outwards exponentially in time (with half the growth rate). As a result, you will eventually reach a time where there is no overlap of individual cells, at the edge of the microcolony, between successive frames. This failure to overlap causes tracking errors. By increasing the frame rate you can increase the size of the microcolony that can be analyzed before these tracking errors occur.

Optimizing the frame rate. Optimizing the data for pipeline analysis implies balancing these two types of errors, one that grows with frame rate and the other that decreases with frame rate. If only the first four or five rounds of division are captured (or analyzed) we often choose a frame rate of 1 / (5 min) or 1/(2 min). However, if we want to track the growth for longer periods, we often use frame rates as high as 1/ (1 min) or 1 / (0.5 min).

5. Reducing segmentation errors using frame skip.

There are a number of experimental applications where very high frame rates are required (e.g. tracking rapid motion). To make these analyses tractable in the OmniSegger (and SuperSegger) pipelines, we implemented a feature called frame skip. Setting the frame skip to n causes the segmentation analysis to segment every n+1 frames. In between these segmented frames, the pipeline copies the masks from the last segmented frame. This feature both speeds up analysis as well as leads to potentially large reductions in fatal errors.

6. Reducing segmentation errors using mask registration.

OmniSegger also has an additional feature to reduce fatal errors which is enabled by default. The linking algorithm checks for 1-to-2-to-1 (and 2-to-1-to-2) errors. If found, a correction algorithm then fuses (fissions) the errant intermediate-frame mask by registering the errant mask to the mask of the previous frame, to maintain the mask shape while approximating the correct mask boundaries. This correction algorithm is not always successful, for example, 2-to-1-to-1-to-2 errors may not always be fixed.

7. Computational resources

Analyses were performed with the following:

- OS: Ubuntu 24.04.1 LTS
- CPU: Intel(R) Core(TM) i9-9900K
- GPU: NVIDIA GeForce RTX 3090 Ti
- RAM: 32 GB

CellProfiler run on Windows 11 with Intel(R) Core(TM) i7-1165G7.

8. Computational runtime

Using the computer described in the Computational resources (Appendix E7), we measured the runtime of the different time-lapse analysis suites. We tested each analysis suite on the normal morphology dataset which was used for the Proliferation challenge. Since CellProfiler required Ilastik preprocessing and was run on a Windows computer, we did not include its runtime. The runtime of each analysis suite is as follows:

- DeLTA - 1m 14s
- OmniSegger - 3m 3s
- SuperSegger - 4m 27s

9. Image processing

The following commands/protocols were used for the analyses in this study:

- DeLTA - Command line
  `delta run -c 2D -i /dir/name/pos{p}cha{c}fra{t}.tif`
- Ilastik/CellProfiler: Followed protocols detailed on Youtube CellProfiler Tutorial: pixel-based classification with ilastik and at ImageProcessing-Benchmarking GitHub [17]
- SuperSegger - MATLAB
  `processExp('dir')`
- OmniSegger - MATLAB
  `processExp('dir')`
