# Supplementary material for: OmniSegger: A time-lapse image analysis pipeline for bacterial cells
Source: PLoS Comput Biol. 2025 May 28;21(5):e1013088. doi: 10.1371/journal.pcbi.1013088 (PMC12140430; doi:10.1371/journal.pcbi.1013088)
Supplement: Attachment — Submitted filename: OmniSegger Response to Reviewers.pdf [file pcbi.1013088.s003.pdf]

Response to Reviews

The authors would like to thank the reviewers for their detailed reading of the manuscript and their many suggestions. In all cases, the reviewers had really substantive comments, which we believe helped to significantly strengthen the story. We sincerely thank them for their time and thoughtful responses to our manuscript. We are optimistic that our responses and significant revisions to the manuscript will satisfy all the reviewers.

Point-by-point: For clarity, we have included the reviewer **Critiques** in black. We have added a single set of numbers to the critiques to allow us to reference them in the responses. **Responses** are in purple and Specific revision **Actions** are in blue. Revised text in the manuscript and Supplementary Material are in red. We have included explicit positions of edits in many cases with line numbers. M = manuscript. SI = Supplementary Material/Information.

Additional Editor Comments:

The reviews for your manuscript are now in hand. Two of the reviewers focused on specific recommended changes to improve content and clarity. The third (reviewer 3) expresses significant concern about the level of innovation, a concern that I share. This is the most important issue to be addressed if you decide to submit a revision especially in view of the journal policy that "Enhancements to existing published methods or software will only be considered if those enhancements bring exceptional new capabilities" (https://journals.plos.org/ploscompbiol/s/journal-information). Addressing the point raised by reviewer 1 about fairness of the comparison to other methods is also important. If resubmitting, please submit a detailed response to all issues raised.

Response: Although many innovative ML-based segmentation tools have been introduced recently, there currently does not exist another robust, comprehensive cell time-lapse analysis suite for analyzing bacterial cells. The OmniSegger package offers such a great increase in performance in general, and in particular in the context both of the analysis of cells with non-canonical morphology as well as the analysis of a range of imaging modalities as to justify a new name and publication. The bottom line is that OmniSegger facilitates analyses of experiments which were not previously tractable.

Reviewer #1: This paper introduces a pipeline of segmenting and analysing time lapses of growing bacterial micro-colonies (OmniSegger), which combines and improves tools of segmentation (OmniPose) and lineage tracking (SuperSegger) from the same authors. The pipeline allows the extraction of single-cell properties as a function of time including dimensions, intracellular localization of proteins, and other features. Segmentation works with different imaging modalities (phase contrast, bright field, and fluorescence), with qualitatively differences depending on contrast and noise. According to a test against two powerful alternative methods (Delta, Cellprofiler), the authors show superior segmentation and lineage tracking based on their own experimental data. The paper is well written and the tool is well described on github. This method should be very useful to a broad community of researchers studying bacteria at the

single-cell level. However, I have a few comments and questions, notably regarding the comparison with alternative methods:

1. Comparison between methods: Did the authors train Omnipose on the same type of data used for the segmentation challenge (i.e., their own data), while the other two methods from different labs (Delta, Cellprofiler) were not retrained? It would then not be surprising that the other two methods perform less accurately. In any case, the authors should add information on training and possibly compare results from their data with results on data from other labs (like the data from the Ingalls lab (ref 21)).

Response: We agree that the distinction about training data could have been more clear in the manuscript. We specifically chose the datasets for this challenge in the OmniSegger manuscript to be datasets new to Omnipose (ie, has not been used in the training data).

Action: We have added a sentence to each challenge (Proliferation, Morphology, Modality) addressing this concern. (M. p.6, 8)

2. Can the authors explain the results recently published by Ingalls (their ref 21), who find Delta more accurate than Omnipose for similar phase-contrast images? See my previous point.

Response: Although we feel that Ingalls paper makes significant contributions, we disagree with these specific results. The Ingalls analysis relies heavily on IOUs to compute performance which is dependent on knowing the ground truth. How is this ground truth determined in the context of phase contrast images? Ingalls offers no clear explanation and therefore this metric of performance is highly subjective.

Although we have previously used fluorescence imaging to independently determine the ground truth, we have avoided this issue entirely in the context of this paper. As we have described already, we consider two distinct error metrics which avoid the subjectivity of previous analyses.

The superiority of OmniSegger in the current context is not difficult to observe, even at a qualitative level. We invite the reviewers to (i) simply inspect the quality of the masks in Fig. 2A generated by competing packages. If quantitation is desired, (ii) consider the cell width histograms shown in Fig. 2D compared to the true cell width, or if a single number is desired, (iii) consult Table II. DeLTA generates a -27% bias in cell width. While DeLTA has strengths, as described in the manuscript, this is NOT good performance. If you use a metric that suggests that it is, there is a serious flaw in the metric.

Action: We did expand our discussion of the flaw with the IOU metric in the paper (M. p.5).

3. Accordingly, the sentence ‘Although some competing pipelines can theoretically generate comparable single-cell cytometry, … [9, 12, 21], their poor segmentation performance limits their applicability.’ and similar statements throughout the results section appear too strong right now.

Response: We disagree with the reviewer. As we demonstrate in the various challenges of our manuscript, the performance of alternative algorithms result in errors which would significantly impede the analysis of a time-lapse experiment. Some of these errors would cause the analysis to be impossible, depending on the analysis.

Consider two practical examples: (i) When we captured the dynamic localizome in E. coli, we analyzed many proteins that were localized to the membrane. At the time, we were using our SuperSegger package which had a cell width bias of -17%. This is large enough to miss a significant fraction of membrane fluorescence! However, since we understood the package intimately, having written it ourselves, we were able to automatically compensate the cell masks in that analysis. It is important to note that using the unmodified masks led to a FAILED analysis. (ii) We have also analyzed essential gene knockouts in Acinetobacter baylyi. These single-cell analyses of growth are simply NOT tractable with competing packages since their performance is so poor. Typically multiple corrections are required per cell in the late phases of growth.

Action: We have added an explanation in the manuscript. (M. p.10,11)

Further comments:

4. SI p4: Bactrack allows the option to use one of three different MIP solvers . What are MIP solvers? Can the authors add a sentence or two?

Response: We agree with the author that this section could be expanded.

Action: We have included more text about the MIP problem and the solvers in the SI. (SI. p.4)

5. I didn't quite understand whether OmniSegger uses by default a segmentation error correction for accurate lineage tracking, or if this is an add-on you actually don't recommend using. The main text does not mention the error correction, and the SI is not very clear on which method is used. Are the data presented in Fig. 2C generated with error correction? Above which frame rate should a user consider error correction necessary, and which method would you then recommend, or do you simply not have a reliable solution for high frame rates?

Response: Regarding "default segmentation error correction": Good question.

Action: We added an additional section on error correction methods to the Supplementary Material, and we call out this additional section in the manuscript. (M. p.6, SI. p.2)

Response: Regarding "Solution to high frame rates: Good question.

Action: We added an additional section on frame rates to the Supplementary Material. (SI. p.7)

Reviewer #2: This is a useful description of a natural progression of a valuable suite of tools. The results are compelling, and the manuscript is well-written. In particular, the attention to

non-typical morphology and to alternate imaging modalities are welcome contributions on important topics that have received relatively little attention.

Some specific comments on the presentation:

6. There's no mention of runtime in the comparisons. These would be a useful addition.

Action: We have included the computing time required for the typical morphology dataset used for the proliferation challenge in the SI Sec. E5. (SI. p.8)

7. In the discussion of 'width' and the comparison to an average in Figure 2D, you may want to add a few words to clarify how width data was collected (to save the reader of the trouble of consulting Appendix C). The description 'inferred from cell contact' is not very informative. In particular, the comparison in Figure 2D seems incomplete (why not compare with a distribution?) until the details in Appendix C are consulted.

Response: We agree.

Action: We have expanded the description of the performance metrics and their rationale. (M. p.5-6)

8. Likewise, on page 8, the statement "However, we note that this challenge underestimates the advantages of phase-contrast over the competing modalities." Seems unjustified until the reader gets to the discussion. Perhaps could add 'as detailed in the Discussion below'

Response: We agree.

Action: Changed. (M. p.9)

Specific wording suggestions:

9. Page 1: "we have attempted to make the algorithms modular…"
This wording leaves open the question of whether you were successful. Perhaps better as "we have made the algorithms modular…"

Response: Thank you!

Action: Changed. (M. p.3)

10. Page 3: "The original segmentation predicted binary masks for each cell (semantic segmentation);…"
Perhaps specify for clarity: "SuperSegger's original segmentation…"

Response: We agree.

Action: Changed. (M. p.3)

11. Page 3: “This file contains > 80 descriptors on a per-cell basis and > 20 on a per-cell per-time-point basis, and is easy to expand by the user.”
‘To expand’ is unclear here: I presume this means the user can incorporate additional descriptors.

Response: We agree.

Action: We have rewritten this sentence to be more clear. (M. p.3)

12. Typos:

Page 1 ‘provide a*n* open-source”

Page 3: “Each of these tools is provided as *a?* MATLAB function

Response: Thank you!

Action: Fixed. (M. p.1,3)

Reviewer #3: In this manuscript, the authors have integrated two tools they have previously developed: SuperSegger, a time-lapse movie analysis pipeline, and Omnipose, a deep learning-based segmentation tool. The combined tool, OmniSegger, leverages the increased performance of Omnipose to fully analyze microscopy movies of coliform bacteria growing on agarose pads. The authors also adapt Omnipose to new imaging modalities, namely brightfield and fluorescence imaging. They show that their software outperforms all similar tools currently available. OmniSegger allows users to fully leverage the power of Omnipose in data analysis and promises to be useful to the community. The manuscript is well written and provides extensive details about the software implementation.

13. However, the manuscript does not demonstrate clear contributions in terms of originality and innovation. The main performance improvements seem to stem from the enhanced segmentation accuracy of Omnipose, while enhancements over SuperSegger’s functionalities such as data visualization, length measurement, and accessibility appear incremental.

Response: The goal of the paper is to share a highly-featured and complete bacterial analysis pipeline with the research community. As we discuss in the paper, this package facilitates analyses of data that were not previously tractable. We believe that users will find that the performance and features of the package will speak for themselves

That said, the reviewer is correct in the sense that most of the gains described are due to the use of our Omnipose package as the segmentation engine. Pragmatically speaking, using Omnipose alone for these same analyses would require many of the tools that we have added in the pipeline. A significant amount of work went into integrating the packages together as well as adding many additional new features. We are pretty open about this in the manuscript. (For instance, see “Omnipose versus Omnisegger”.)

We believed it was important to publish the pipeline as described here for simplicity and clarity for readers and users. This is the package that we use as the basis for our quantitative image analyses.

Action: We modified the abstract language to propose OmniSegger more clearly. (M. p.1)

Minor Comments:

14. Consider moving panels B-F from Figure 1 to later sections, or creating a Supplementary Figure, to discuss Cytometry features and the data exploration GUI in more details. A table listing all cytometry features should be added to the Supplementary Material, highlighting new or modified features compared to SuperSegger. Figure 1D should be enlarged.

Response: We did consider both organizations when writing the paper. In the end, we believe that the organization as presented is strongest.

Because OmniSegger is a data analysis suite capable of various measurements and visualizations, we feel that the first figure should highlight its uses for prospective readers. We therefore chose to show a sample analysis that is highly characteristic of the experiments in our lab. The most significant new addition to the cytometry measurements is the addition of a cell length and cell width measurement, which assumes a rod-shaped geometry. The details of this measurement are included in the SI. We have not added any other significant cytometry measurements as the original cytometry measurements performed by SuperSegger are very comprehensive for the experiments done in our lab.

There is also a subtle issue about "what features are novel" with regard to our two previous packages SuperSegger and Omnipose. We were worried that readers might find the presentation dishonest if we positioned features that already existed in our previous pipeline at the end of the paper. Of course, the increased performance of the upgraded package makes these existing features much more powerful in the new OmniSegger package.

Action: None.

15. The authors should provide details on the computing time required for one time-lapse movie and compare this with other available tools.

Action: See 6.

16. It is unclear whether the tracking/linking performance of the method was evaluated, in addition to the segmentation performance metrics such as cell width and fatal errors.

Response: Pipeline performance as measured by "fatal errors" depends on both the single-frame segmentation and cell linking.

Action: We have included new text clarifying this issue as well as explaining the rationale for our approach. (M. p.6)

17. From the data availability section, it is unclear if the Fluorescence Omnipose models will be made available on Zenodo.

Response: We agree that this could have been more clear. The fluorescence Omnipose model was available with the release of Omnipose and is already included when installing Omnipose (bact_fluor_omni).

Action: We have added a sentence that addresses this in the data availability. (M. p.11)

18. Page 8: "However, we note that this challenge underestimates the advantages of phase-contrast over competing modalities" --> Add "(see Discussion)" or remove this sentence.

Response: We agree.

Action: We have included this change. (M. p.9)

19. In Figure 6, please include y-axis values.

Action: We have changed the figure to include numbers on the y-axis. (M. p.11)
